# The Influence of Providing and Receiving Social Support on Older Adults' Well-being

**DOI:** 10.2174/17450179-v18-e2112241

**Published:** 2022-02-03

**Authors:** Nasibeh Zanjari, Yadollah Abolfathi Momtaz, Seyed Hossein Mohaqeqi Kamal, Mehdi Basakha, Sina Ahmadi

**Affiliations:** 1 Iranian Research Center on Aging, University of Social Welfare and Rehabilitation Sciences, Tehran, Iran; 2 Malaysian Research Institute on Ageing (MyAgeing), Universiti Putra Malaysia, Serdang, Selangor, Malaysia; 3 Department of Social Welfare Management, University of Social Welfare and Rehabilitation Sciences, Tehran, Iran

**Keywords:** Providing social support, Receiving social support, Well-being, Older adults, Cognition, Demographics

## Abstract

**Introduction:**

Social support is a leading contributing factor for older adults' well-being. The present study aimed to compare the impact of two-way (providing and receiving) social support on the well-being of Iranian older adults.

**Methods:**

The present cross-sectional study was conducted on 1280 community-dwelling older adults in Tehran, Iran, 2020. The researcher used the clustered sampling method and the 2-way Social Support Scale (SSS) to collect samples and measure social support, respectively. The well-being was measured by the self-reported World Health Organization-Five Well-Being Index (WHO-5). Bivariate and hierarchical linear regression analyses were performed to compare the effects of social support aspects on well-being. Data were analyzed using SPSS 20.0. A significance level of p≤0.05 was considered statistically significant.

**Results:**

The mean age of the respondents was 70.90 (SD=8.07), and about 70% of the sample was married. The mean scores of taking and providing social support were 20.70 ±7.52 and 17.71 ±7.82, respectively. The hierarchical regression analysis revealed that providing social support is significantly associated with the well-being of older adults beyond and over receiving social support and possible contributing factors (∆F=30.25; ∆R2= 0.39, p<0.05).

**Conclusion:**

The results showed that providing social support is more important than receiving it. Older adults should participate in social activities to provide social support.

## 1 INTRODUCTION

Most countries worldwide have been experiencing increased life expectancy and low fertility, leading to the prevalence of aging. According to the United Nations report, in 2020, there were 727 million older people aged 65 years or over, and older people are projected to get double by the year 2050 [1]. Moreover, in Iran, this population is increasing rapidly. According to the 2016 census results, 9.3% of the total population of Iran were aged 60 years and older, and it is projected to rise around 32% by 2050 [2]. Changes in the household composition and living arrangement highlight the importance of social support and social networks.

Social relationships and social support are essential for well-being in all age groups. Social support becomes more necessary since individuals encounter challenges (empty nest syndrome, social isolation, and morbidity) while getting old. Psychosocial determinants (social engagement and social support) have a prominent role in older adults' well-being and improve their quality of life [3, 4].

The primary model of successful aging (proposed by Rowe and Kahn) [5] states that active engagement includes social support, activities, and capability of maintaining physical functioning, leading to healthy and successful aging. Moreover, social support protects them against the adverse effects of stressful life events, such as diseases and bereavement. The presence of someone to assist in later life protects individuals from aging losses [3, 6].

The association of social support with mental or physical health has been well documented. A nine-year follow-up study involving 6928 residents of Alameda County revealed that participants who lacked social ties were more likely to die in the follow-up period [7]. A review of 81 studies showed that higher social support is correlated with better cardiovascular function and immune system. This finding indicated the critical role of emotional support and familial sources of support as a potential mechanism [8]. MacArthur's cohort study on 1,189 older adults revealed that receiving more emotional support was a significant predictor of better cognitive function during the 7.5-year follow-up [9].

Moreover, a systematic review of 39 studies stated the positive association of social support with global cognition and episodic memory [10]. Recent studies have focused more on the different aspects of the quality of social support on health. A cross-sectional survey on the 1146 German older adults indicated that the quality of kin and non-kin support positively correlates with elderly well-being. However, emotional support from kin and instrumental support from non-kin contribute to well-being. It means that a good relationship could moderate the negative impact of receiving instrumental support from family members [11]. A systematic review of 66 studies highlighted the importance of family social support in Asia than in western countries. Furthermore, the present study has indicated the positive effect of good social, instrumental, and emotional support on decreasing depressive symptoms among Asian community-dwelling older adults [12]. Thus, it can be concluded that social support protects senior adults from stress and depression [13].

Different theories and models may explain the underlying mechanisms of the impact of social support on the quality of life in later life. These theories include Convoy theories, Socioemotional Selectivity Theory (SST), Strength and Vulnerability Integration Model (SAVI), and functional specificity theory. The idea is that older adults' networks consist of the family rather than friends, and the variety of the social network correlates with higher well-being. Convoy theory states that all aspects of social relationships are mutually influential; that is, members of a social network influence each other [4, 14]. According to the SST, older adults focus more on relationships by selecting close and emotional connections due to the time limitation. Older people need to spend time with those who are helpful for their well-being. Time and age affect the meaning of life and whether or not to receive emotional support [15, 16]. The SAVI model emerged from SST and suggested that older people can improve coping skills and avoid negative interpersonal experiences. Aging reduces physiological flexibility, increases life perception, and contributes to adaptation to losses [17]. The Functional Specificity Theory indicates the different emotional support individuals receive as a function of relationships to maintain individual wellness [4].

Other psychosocial theories or models (self-determination theory, generativity, and identity theory) explain the role of social health support [18]. Self-determination theory states that providing support to others satisfies basic human needs (including autonomy, competence, and relatedness) [19]. Moreover, Erikson's development theory states that sharing knowledge and experience promotes ego integrity in later life [20]. Identity theory indicates that receiving support may reduce the competence and disturb the identities of older adults, which is consistent with previous theories [18].

From the social perspective, reciprocity is a cultural norm. This viewpoint emerges in social exchange theory [20], which shows the significant role of cost-benefit in interpersonal relationships. Social support is not one-way assistance; it is a process of social exchanges. However, informal social support among family members is a viable part of life. It is applicable in intergenerational relationships, support systems, and informal care [21, 22].

Assessing social support relies on the conceptualization of this concept. Most of the studies measure social support in terms of its functional component. Accordingly, social support constitutes emotional support (caring and love), instrumental support (tangible support), informational support (guidance and providing solution), appraisal support (help for self-evaluation), and social companionship (spending time for recreation) [14, 23]. Some studies have measured social support based on the number of supportive people in their social network [24].

These theories focus on being loved and valued by others but do not address the person's perception of helping others [25].

Socio-demographic groups discuss the direct and indirect effects of social support on health. Older women receive more support due to a more extensive network than men. Older men receive more support from their wives and report more satisfaction. In contrast, older women receive support from their children or friends [26]. Receiving social support is important among older patients as it helps them recover from illness, injury, and health maintenance.

Moreover, social support helps manage diseases and promotes adherence to treatment or medicine [27]. However, some studies revealed the negative effect of receiving too much social support on well-being [28]. Thus, the quantity and quality of the received support are challenging and questionable. Many factors such as low income, loneliness, disease, or dependency on daily activities can affect the quantity and quality of the received support [29].

Recently, several studies have assessed the reciprocity nature of social support. Some of these studies reported that providing social support to older people is as important as receiving it [30, 31]. These studies had some limitations. Few studies examined the effect of provided and received instrumental and emotional support on well-being. The present study examined the two-way (reciprocal) social support effect on the well-being of Iranian older adults.

## 2 METHODS

### 2.1 Participants and Data Collection

This cross-sectional study was performed in Tehran in 2020. The population consisted of community-dwelling individuals aged 60 years and older. A sample of older adults (N=1280; mean age: 70.90 (±8.07); female: 70.10 (±7.86), male: 71.70 (±8.20)) was selected by the clustered sampling method in 22 districts of Tehran. The sample size (for each district) was estimated based on a percentage of old residents. Two neighborhoods were randomly chosen in each district—older adults living there had face-to-face interviews. Interviews ranged from 10 to 20 minutes. The inclusion criteria included being 60 years and over, being defined as community-dwelling older people living in Tehran for at least one year at the time of data collection, willing to participate in the study, and providing oral consent. The exclusion criteria included providing incomplete answers to questionnaires, vague answers due to mental instability, or being deaf and dumb.

The present study has taken approval from the Research Ethics Committee of the University of Social Welfare and Rehabilitation Sciences (Ethical code: IR.USWR.REC.1398.068).

### 2.2 Instruments

#### 2.2.1 The Brief 2-Way Social Support Scale (SSS)

The SSS [3] measures social support. The SSS was originally developed by Shakespeare-Finch, Obst [32] with 20 items and a brief version of 12 items. The psychometric properties of the concise version provide evidence for the scale to be reliable with 12-items in 2020. The SSS evaluates the provided and received social support based on instrumental and emotional support. In the present study, Cronbach's alpha coefficients were 0.92 and 0.94 for receiving and providing social support, respectively.

#### 2.2.2 The World Health Organization- Five Well-Being Index (WHO-5)

The WHO-5 [33] was employed to measure well-being. The WHO-5 is a standard and short scale measuring subjective well-being. It is scored from 5 (all of the time) to 0 (none of the time) and ranges theoretically between 0 to 25. The WHO-5 found a high internal consistency in the present study (Cronbach's alpha=0.91).

Moreover, possible variables included sex, age, marital status, level of education, number of children, living arrangements, perceived socioeconomic status (SES), being independent for daily activity at home or out of the house, and perceived health status.

### 2.3 Data Analysis

Descriptive and inferential analyses were done using SPSS 20.0. Descriptive statistics illustrating the study sample's characteristics included percentage, frequency, and mean. The inferential statistics included one-way ANOVA, independent samples t-test, and multiple hierarchical regression analysis. It determined the relations between social support dimensions and well-being with and without controlling demographic variables. A significance level of p≤0.05 was considered statistically significant.

## 3 RESULTS

Around 50% of respondents were women in the present study. The mean age of the participants was 70.90 (SD=8.07). About 70% of respondents were young-old individuals. Moreover, 13.4% of the sample had no formal education.

Table **1** displays bivariate analyses showing a statistically significant association between demographic and characteristics variables and well-being (except for sex and the number of children).

Table **2** displays mean social support scores; the mean score of receiving social support (M=20.70 ±7.52) was more than providing social support (M=17.71 ±7.82).

Hierarchical regression analysis was conducted to compare the influence of providing and receiving social support on well-being. First, the assumption multicollinearity was assessed based on the correlation between all variables in the regression analysis. Table **3** shows no evidence for multicollinearity.

Table **4** shows the hierarchical regression analysis in three blocks to compare the influence of receiving and providing social support on well-being, controlling the demographic and individual variables.

Model 1 shows demographic variables that significantly influence well-being. This block shows a significant overall model (F=15.52, P<0.001), explaining around 11% of the variance in well-being.

Model 2 includes receiving social support. Instrumental social support was significantly associated with well-being after controlling demographic and individual variables (p<0.001), as shown in Table **4**. This significant model (F= 18.163, p<0.001) explained an additional 3% of the variance in well-being beyond the demographic and individual variables.

The provided social support was added in block 3. The results of model 3 revealed that emotional and instrumental provided social support significantly contribute to well-being, above and beyond receiving social support, and possible confounding variables (∆F=30.25, ∆R^2^= 0.39).

## 4 DISCUSSION

The present study on Iranian older adults aimed to investigate the influence of provided and received instrumental and emotional social support on well-being. The mean score of receiving social support was higher than providing it. Although receiving instrumental support is associated with improved well-being, it loses its significant positive correlation with well-being when examined simultaneously with provided instrumental and emotional social support.

Previous studies have reflected the importance of receiving or seeking social support among older adults. However, the present study highlighted providing social support even in later life. Providing instrumental social support is strongly correlated with higher well-being. The finding revealed that providing tangible or instrumental support for others improved well-being in later life.

Providing social support illustrates the independence and higher socioeconomic status of older adults. This result is consistent with other studies indicating the instant benefit of providing social support than receiving it [32, 34], which results in decreased depressive symptoms [35].

Receiving social support is more critical in traumatic situations (diseases or feeling depression or anxiety) [31]. However, being independent and helping others increase self-esteem, engagement with life, and aging well [25]. The present study results showed that those who are independent in performing daily activity out of the house have higher perceived SES, better health status, and improved well-being. Identity theory explains the highlighted role of providing social support. It can foster the feeling of independence and usefulness to others and actualize older adults' identities [36].

Providing social support reduces stress-related mechanisms and increases reward-related activity. These neural mechanisms impact health and well-being [18]. Successful agers attempt to engage more in life and provide support to others. It helps them to adapt to aging losses. As Iranian elderly are family-oriented, they support their children, thus promoting their well-being. Although the present cohort of old Iranian people has adequate children (the mean number of children is five) to receive support, they keep their authority by providing support to promote their well-being and sense of competence [37].

As a type of intergenerational capital, providing and receiving support can be differently perceived by people from various cultures and societies. In Iran, where the leading social support resource is family, dignity and respect are highly prevalent.

According to Erikson's development theory, “generativity” results in ego integrity and successful aging. Generativity is not just raising children; it also includes volunteering and mentoring activities or caring for others providing social support. If individuals do not engage in productive deeds, they may experience stagnation and unproductiveness [38]. Providing social support as generativist creates a feeling of helpfulness in their community.

Our results align with social exchange theory [39]; the tangible and intangible resources of social support exchanging between older people and other social actors are based on the quality and quantity of receiving and giving rewards and costs. Receiving more support leads to unequal power between social actors (older adults and others) and emotional elder abuse.

Young people believe that the more support they provide, the more they get when aged [40]. However, reciprocal social support communal relationship is not defined as a business connection. Providing tangible support does not anticipate receiving a comparable benefit [41]. Maintaining intergenerational solidarity or being helpful can be the promising benefits they receive in later life, leading to well-being.

Providing support is beneficial if individuals (such as older adults) freely offer help and support to others [40, 41] and feel connected in their social relationships. Moreover, providing support and caring for offspring, partners, other family members, or friends is rewarding, thus increasing self-worth and belonging to a social network.

## CONCLUSION

In summary, the present study confirms that providing social support is more important than receiving it for the well-being of older adults. So, facilitating the intergenerational social support exchange or volunteering in social activities could help older adults maintain their authority and independence in their social relationships and consequently feel well in later life.

## LIMITATION AND SUGGESTIONS

Due to the limitation of the cross-sectional survey, we were unable to investigate the causal effect of social support on well-being. Further studies need to use a longitudinal design to examine this relationship. Furthermore, it is recommended to measure the impact of social network structures regarding people who receive support from older adults and its quantity on their well-being.

## Figures and Tables

**Table 1:** The means of well-being by demographic variables.

**Variable**	**Category**	**%**	**Mean(SD)**	**F / t**
Age groups	young old	70.2	15.47(5.56)	16.870***
	old	22.5	13.98(6.00)	
	oldest-old	7.3	12.45(6.25)	
Sex	men	50.1	14.97(5.93)	0.713
	women	49.9	14.85(5.65)	
Marital status	unmarried	29.8	13.43(6.13)	-6.062***
	married	70.2	15.54(5.52)	
Education	illiterate	13.4	13.39(6.31)	9.781***
	informal education	11.2	13.13(6.24)	
	primary	18.2	15.26(5.70)	
	secondary	17.2	14.35(5.45)	
	diploma	20.8	16.20(5.38)	
	higher education	19.3	15.80(5.44)	
Number of children	0	3.3	13.95(5.82)	1.181
	1	5.2	15.88(5.67)	
	2	21.6	14.86 (5.95)	
	3	26.3	15.19(5.60)	
	4	20.5	14.65(5.59)	
	5	12.2	14.29(6.21)	
	6+	10.9	15.37 (5.84)	
Living arrangement	alone	12.4	13.60(6.17)	
	not alone	87.6	15.10(5.71)	-3.074***
Perceived SES	low	17.1	12.87(5.30)	17.105***
	middle	66.6	15.28(5.60)	
	high	16.3	15.55(6.57)	
Independent for daily activity at home	yes	92.7	15.18(5.67)	5.828***
	no	7.3	11.61(6.27)	
Independent for daily activity out of home	yes	74.4	15.65(5.49)	7.947***
	no	25.6	12.77(6.11)	
Perceived health status	bad	10.4	11.89(6.15)	47.304***
	fair	30.5	13.62(5.88)	
	good	59.1	16.11(5.33)	

Note: * p≤0.05, ** p<0.01, ***p<0.001

SES: socioeconomic status; SD: standard deviation.

**Table 2:** The mean score of social support.

**Variables**	**M(SD)**	**95% CI**
Total RSS	20.70(7.52)	[20.26, 21.09]
Total GSS	17.71(7.82)	[17.29, 18.12]
Instrumental RSS	10.64(4.22)	[10.40, 10.86]
Emotional RSS	9.08(4.14)	[8.86, 9.31]
Instrumental GSS	10.06(3.79)	[9.84, 10.26]
Emotional GSS	8.63(4.20)	[8.41, 8.86]
Well-being	14.91(5.79)	[14.59, 15,22]

SD: standard deviation; M: mean; CI: confidence interval; GSS: giving social support; receiving RSS: social support

**Table 3:** Results of the pearson product-moment correlation coefficients.

	2	3	4	5	6	7	8	9	10	11	12	13	14	15
1.Well-being	-0.01	-0.17**	0.17**	0.16**	-0.01	0.09**	0.14**	0.26**	-0.16**	-0.21**	0.32**	0.29**	0.21**	0.17**
2. Sex		-0.09**	-0.27**	-0.26**	0.00	-.14**	-.07**	-.09**	.12**	.19**	0.03	.07**	0.05	0.03
3. Age			-0.26**	-0.29**	0.19**	-0.06*	0.00	-0.34**	0.38**	0.50**	-0.16**	-0.14**	0.01	0.01
4. Marital status				0.34**	-0.06*	0.56**	0.08**	0.15**	-0.26**	-0.25**	0.22**	0.18**	0.17**	0.15**
5. Education					-0.35**	0.15**	0.31**	0.22**	-0.27**	-0.36**	0.16**	0.11**	0.12**	0.10**
6. Number of children						0.08**	-0.18**	-0.16**	0.14**	0.18**	0.06*	0.10**	0.10**	0.06*
7. Living arrangement							0.01	-0.03	.07*	0.00	0.17**	0.16**	0.20**	0.17**
8. Perceived SES								0.21**	-0.09**	-0.12**	-0.02	0.03	0.11**	0.20**
9. Perceived health status									-0.41**	-0.51**	0.09**	0.06*	-0.06*	-0.02
10. Independent for daily activity at home										0.47**	-0.11**	-0.07**	0.05	0.03
11. Independent for daily activity out of home											-0.07**	-0.05	0.10**	0.03
12. Instrumental GSS												0.75**	0.65**	0.48**
13. Emotional GSS													0.71**	0.60**
14. Instrumental RSS														0.76**
15. Emotional RSS														

Note: * p≤0.05, ** p<0.01, ***p<0.001

SES: socioeconomic status; GSS: giving social support; receiving RSS: social support

**Table 4:** The influence of receiving and providing social support on the well-being of older.

adults									
**Predictors**	**Model 1**			**Model 2**			**Model 3**		
	**b**	**Beta**	**t**	**b**	**Beta**	**t**	**b**	**Beta**	**t**
Sex	.813	.070	3.162***	.524	.045	1.557	.384	.033	1.165
Age	-.023	-.031	2.383	-.022	-.031	-.938	-.006	-.008	-.239
Independent for daily activity at home (Yes)	-.296	-.013	-.935	-.376	-.017	-.533	.028	.001	.041
Independent for daily activity out of home (Yes)	-.979	-.074	-.411***	-1.358	-.102	-2.866*	-1.209	-.091	-2.607*
Marital status (unmarried)	1.060	.084	-2.038***	.761	.060	1.693	.537	.042	1.218
Education	.249	.071	2.320***	.118	.034	1.028	.080	.023	.715
Number of children	.330	.086	2.148***	.214	.056	1.943*	.162	.042	1.495
Living arrangement (alone)	.593	.034	2.965	.179	.010	.311	.204	.012	.360
Perceived SES	.778	.078	1.011***	.577	.058	2.002*	.947	.095	3.311*
Perceived health status	1.562	.183	2.702***	1.623	.190	5.976*	1.406	.164	5.263*
Emotional RSS				-.018	-.013	-.318	-.030	-.022	-.543
Instrumental RSS				.330	.216	5.219*	.012	.008	.167
Emotional GSS							.126	.090	2.013*
Instrumental GSS							.311	.225	5.422*
-	F= 15.52*** R=0.33, R^2^=10.9% ∆ R^2^= 0.109 ∆F= 15.52***			F= 18.163*** R=0.38, R^2^=14.7% ∆ R^2^= 0.038 ∆F= 28.06***			F= 20.608*** R=0.43, R^2^=18.6% ∆ R^2^= 0.39 ∆F=30.25***		

Note: * p≤0.05, ** p<0.01, ***p<0.001

SES:socioeconomic status; GSS: giving social support; receiving RSS: social support

## Data Availability

The data sets used during the current study can be provided from the corresponding author [Y.A.M], upon reasonable request.
